# Metabarcoding and targeted barcoding can enhance Norwegian Continental Shelf macrofauna species inventories

**DOI:** 10.7717/peerj.20849

**Published:** 2026-02-20

**Authors:** Jon T. Hestetun, Anders Lanzén, Jon Anders Kongsrud, Tom Alvestad, Per-Otto Johansen, Thomas G. Dahlgren

**Affiliations:** 1 Climate and Environment, NORCE Norwegian Research Centre, Bergen, Norway; 2 AZTI, Pasaia, País Vasco, Spain; 3 University Museum, University of Bergen, Bergen, Norway; 4 Gothenburg Global Biodiversity Centre, University of Gothenburg, Gothenburg, Sweden

**Keywords:** Metabarcoding, Benthos, Biodiversity, Molecular ecology, Invertebrates, COI, 18S, Macrofauna, eDNA

## Abstract

Metabarcoding of bulk community samples is a powerful tool to characterize marine softbottom macrofaunal communities, but high-quality taxonomic assignment is dependent on adequate sequence coverage in taxonomic databases. Here, our main aim was to advance metabarcoding as a complement to benthic morphological taxonomy in biodiversity inventories on the Norwegian Continental Shelf (NCS). We used morphological taxonomy, barcoding, and metabarcoding for two objectives, namely to (1) increase macrofauna barcode coverage for a selection of species, and (2) provide an in-depth comparison of morphology and metabarcoding data from mock bulk samples of softbottom macrofauna. We used morphological taxonomy to identify 257 morphotaxa from 32 sieved grab sampling stations at eight areas on the NCS. For the first objective (barcoding), 45 species (95 specimens) were selected from these 32 stations based on incomplete sequence coverage in online repositories, obtaining barcodes for 25 (cytochrome oxidase subunit 1, COI), 35 (18S rDNA), and 24 (28S rDNA) species. Results typically showed an increase in taxonomic assignment of 4–5 ranks in the subsequent metabarcoding data for these particular species. For the second objective (metabarcoding), mock bulk samples with a known taxonomic composition including Annelida, Arthropoda, Mollusca and a single brachiopod, were sequenced using the COI and 18S rDNA V1-V2 partitions from a subset of eight stations from three of the areas. COI Barcode of Life Data System (BOLD) and MIDORI2 assignment with some additional manual sequence curation recovered 100 distinct species-rank taxa compared to 120 species-rank taxa (152 taxa total) from morphology based taxonomic identification. Assignment of 18S rDNA using SILVA recovered 29 unique species including 13 not found in the COI data. Annelida, Arthropoda, and Mollusca were all well-represented in metabarcoding data, and abundance biases were associated with disparate species in a range of clades. Taxonomic congruence was high at high rank, but in some cases species assignments resolved as genus only or sibling species to those identified by morphological taxonomy even when present in one of the databases used. Potential explanations include species genotype variation, putative species complexes and remnant sequencing artifacts. The study findings show the potential of metabarcoding in an area with relatively high taxonomic database coverage. Integrating metabarcoding datasets can increase biodiversity inventory pace and uncover hidden biodiversity, but performance is dependent on database coverage, highlighting the importance of barcoding efforts in biodiversity studies, and metabarcoding-based inventories need to be critically examined by taxonomic expertise.

## Introduction

Study of marine softbottom benthic macrofauna biodiversity commonly involves the taxonomic identification of sieved benthic samples from sediment grab or box corer samples. Connected to the development of high-throughput sequencing in the last decades, using community-level amplification of genes, metabarcoding, to map organisms in a given sample, have become increasingly common (Gielings et al., 2021). Several studies (Aylagas et al., 2016; Aylagas et al., 2018; Cowart et al., 2015; He, Sutherland & Abbott, 2021; Lobo et al., 2017; Van der Loos & Nijland, 2021) has shown the potential of using metabarcoding from community samples—homogenized bulk specimen samples—as a method for identification of benthic organisms. Metabarcoding has the potential to increase the capacity to map benthic biodiversity patterns, including in vulnerable and poorly known areas such as the Arctic and deep sea (Laroche et al., 2020; Leduc et al., 2019; Murray et al., 2024). Thus, it could help increase the scope and analysis speed for benthic biodiversity surveys and marine impact monitoring, leading to better management outcomes in mitigating current anthropogenic impact on benthic ecosystems (Aylagas et al., 2016; Cordier et al., 2021; Deiner et al., 2017).

While metabarcoding, especially for environmental-DNA (eDNA) samples of sediment or water, often focuses on organisms outside the scope of macrofaunal morphotaxonomic inventories such as micro- or meiofauna, macrofaunal datasets have some unique features compared to that of other organisms: high taxonomic coverage for cytochrome oxidase subunit I (COI), a main marker in metazoan barcoding efforts, in online databases such as MIDORI Reference 2 (Leray, Knowlton & Machida, 2022), the Barcode of Life Data Systems (BOLD) (Ratnasingham & Hebert, 2007) or NCBI GenBank can enable comparisons with existing morphological time series and easier ecological interpretation of metabarcoding data (Andújar et al., 2018; Van der Loos & Nijland, 2021). Notably, several studies have shown that biotic indices from taxa identified from metabarcoding data can produce ecological assessment comparable to currently used >1 mm macrofaunal morphological datasets (Aylagas et al., 2016; Hestetun, Lanzén & Dahlgren, 2021; Lejzerowicz et al., 2015). More broadly, macrofaunal metabarcoding data can be used as a valuable tool in increasing the capacity for baseline characterization, mapping, and species inventories of softbottom habitats, and identifying hidden biodiversity within macrofaunal species and species complexes (Turon et al., 2020).

COI is a protein-coding gene lacking truly conserved primer binding sites. This requires the use of degenerate primers (Kessing et al., 1989; Leray et al., 2013; Wangensteen et al., 2018) to increase the number of taxa that can be amplified using this marker. But even degenerate COI primers exhibit significant and inherent taxonomic biases (Aylagas et al., 2016; Clarke et al., 2014; Deagle et al., 2014; Ren, Zhou & Zhang, 2025), and polymerase chain reaction (PCR) amplification outcomes are dependent on the amount of DNA from easily amplified taxa in the sample, whose relative presence will impact amplification abundances for the entire dataset (Klunder et al., 2022). Secondly, while COI online taxonomic database coverage is comparatively high, there are still large gaps in coverage for also well-known areas, which can limit the extent to which metabarcoding sequences can be identified to lower taxonomic rank (Hestetun et al., 2020).

Another gene that can capture significant metazoan diversity is the 18S rRNA gene. Several metabarcoding primer pairs can be used to amplify part of the 18S rRNA gene sequence, focusing on variable sections of the gene including the V1–V2 or V4 regions (Stoeck et al., 2010; Hadziavdic et al., 2014; Sinniger et al., 2016). These markers tend to produce data that includes both metazoan (including both meio- and macrofauna) and single-celled eukaryote clades. Ribosomal markers have stem-region primer sites that are more conserved compared to COI (Collins et al., 2019), and stem regions allow easier identification of ribosomal sequences at higher taxonomic rank, yet less overall sequence variability makes species-rank identification more difficult (Leray & Knowlton, 2016; Tang et al., 2012) and macrofaunal 18S rDNA database coverage (GenBank and SILVA) is lower than for COI (Hestetun et al., 2020).

A more widespread implementation of infaunal metabarcoding requires more in-depth study of the extent of biases in metabarcoding data from community samples to assess the role and use cases of macrofaunal metabarcoding data in conjunction with morphotaxonomic studies. Benchmarking of metabarcoding data against samples of known morphological composition (mock community) has been done for several marine habitats (Aylagas et al., 2016; Cahill et al., 2018; Lobo et al., 2017; Steyaert et al., 2020; Van der Loos & Nijland, 2021). The level of obtainable taxonomic resolution is, however, entirely dependent on the amount of barcoding effort in a particular area, meaning benchmarking results vary with time and place.

The main aim of this study is to assess and develop bulk community sample metabarcoding as a complement to morphological taxonomy biodiversity assessment in softbottom benthic habitats on the Norwegian Continental Shelf (NCS). The NCS, which spans the Northern North Sea and Norwegian Sea, is an area subject to large-scale sampling of benthic softbottom macrofauna as part of offshore oil and gas extraction monitoring campaigns part of Norwegian environmental regulations (Norwegian Environment Agency, 2020). Extensive time series, stretching back decades, exist for softbottom infauna along the shelf off the Norwegian coast (DNV, 2023), and there has been a handful of recent sediment eDNA studies from the area (Hestetun, Lanzén & Dahlgren, 2021; Lanzén et al., 2021; Mauffrey et al., 2021). As a result of ongoing taxonomic and barcoding effort at institutions in bordering countries and targeted initiatives (*e.g.*, Derycke et al., 2021), the North Sea also has a relatively high taxonomic coverage of macrofaunal benthic species in online sequence databases (Hestetun et al., 2020).

The study is divided into two main parts: (1) The first objective comprises a targeted barcoding effort on macrofaunal species with identified coverage gaps. These species were collected from eight areas in the North and Barents Seas. (2) The second objective—using a subset of three of these eight areas from the North Sea—is a comparative analysis of taxonomic composition between morphological species lists, and COI and 18S rDNA community metabarcoding taxonomically assigned Operational Taxonomic Unit (OTU)/Amplicon Sequence Variant (ASV) tables. Here, we were interested in seeing what was missing in the metabarcoding data, but also if this data could reveal hidden biodiversity in the morphospecies included in the study, *i.e.,* how do these data types complement each other in an integrative study of macrofaunal biodiversity? To this end, we focused specifically on (1) patterns of higher- and lower-rank compositional difference between datasets (*i.e.,* severity and taxonomic rank of COI and 18S rDNA taxonomic biases relative to morphological data), (2) the recovery of identified morphological taxa in the COI and 18S rDNA datasets from the same mock bulk community samples at high and low taxonomic rank, and (3) to what extent the metabarcoding data detected the presence of hidden species diversity in the morphological data. Finally, we interpret our findings to discuss how morphological and metabarcoding data perform as complementary components in integrated studies characterizing and monitoring softbottom macrofaunal communities.

## Materials & Methods

### Sampling

**Figure 1:**
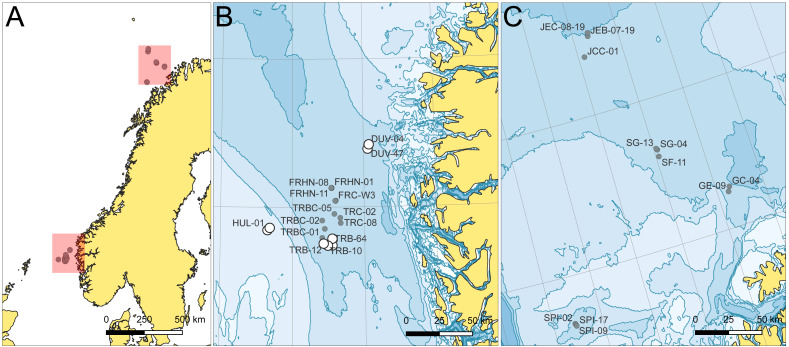
(A) Study areas and sampling stations in the (B) Northern North and (C) Southern Barents Seas. Gray circles denote stations included in the barcoding effort only, while white circles denote stations that were included in both the barcoding and metabarcoding mock bulk community analysis in this study.

The macrofauna samples for this study were collected using 0.1 m^2^ van Veen grabs during two environmental monitoring cruises to the Northern North Sea by DNV (*MV Electron*, May 21–June 1, 2019) (Møskeland, Brooks & Ulfsnes, 2020) and the Southern Barents Sea by Akvaplan-niva (*MV Christina E*, May 21–June 6, 2019) (Mannvik, Wasbotten & Andrade, 2020) as part of the Norwegian offshore benthic monitoring program (Fig. 1). Samples include 32 van Veen grab sampling stations (one grab replicate per station) from eight areas, sieved using one mm sieves on deck: Troll (9 st), Fram (7 st), Huldra (2 st), Duva (3 st), Snøhvit (3 st), Goliat (2 st), Spissa (3 st) and Johan Castberg (3 st). Sieves and equipment were cleaned between each station. The areas are named after local oil and gas surface and subsea installations, but stations with known environmental impact were not included in this study (Mannvik, Wasbotten & Andrade, 2020; Møskeland, Brooks & Ulfsnes, 2020). Except for Huldra (121 m), station depth was between 304–401 m for all stations (Table S1). No ethical approval for animal use was required under Norwegian legislation as sampling included only non-cephalopod invertebrate taxa and no experimental procedures.

All the sampling stations were used to collect potential specimens for barcoding. Sampling was done by sieving the one mm fractions of grab samples from each station and preserving them in 96% ethanol, changed within 24 h of initial preservation. For the second objective of this study, *i.e.,* the comparative metabarcoding study, a subset of eight sampling stations from three areas, Troll (4 st), Huldra (2 st) and Duva (2 st), were used to make mock bulk community samples for high throughput sequencing.

### Morphological identification and barcoding

Morphological identification of specimens was done for all stations in the dataset, *i.e.,* 32 stations from all eight areas. Specimens were separated from remaining sediment and identified by the authors of this study: JK (Annelida), TA (Annelida), POJ (Arthropoda) and JTH (Mollusca, Brachiopoda). Species classification was checked and harmonized with the World Register of Marine Species (WoRMS Editorial Board, 2025). Species lacking either COI or 18S rDNA sequences in online repositories were targeted for barcoding by checking the names of identified species against GenBank, BOLD and SILVA using the method described in Hestetun et al. (2020). The purpose of the barcoding effort here was not limited to the metabarcoding aim of this study but rather thought as part of a general cumulative effort to increase sequence coverage of NCS macrofauna. For this reason, standard barcoding (rather than metabarcoding) primers were employed, and 28S sequences were added to the barcoding effort even though this gene is not part of the metabarcoding part of this study.

For specimen barcoding, DNA was extracted from individual specimens using the Qiagen Blood and Tissue Kit with the QIAsymphony extraction robot (Qiagen, Hilden, Germany). PCRs were done using the KAPA3G Plant PCR Kit (Roche, Basel, Switzerland). Initial COI “Folmer” barcode amplification of polychaetes was done with the polyLCO1490 and polyHCO2198 primer pair (Carr et al., 2011), and mollusks using jgLCO1490 (Geller et al., 2013) and COIschneckrev (Jaksch et al., 2016). Crustacean and subsequent polychaete and mollusk COI amplification (for specimens that did not amplify with the original primer pair) was done using the jgLCO1490 and jgHCO2198 primer pair (Geller et al., 2013). Amplification of partial 18S rDNA was done using the F-40 and R-1196 primer pair (Hadziavdic et al., 2014). Partial 28S rDNA amplification was done using the 285F (Struck, Purschke & Halanych, 2006) and PO28R4 (Passamaneck, Schander & Halanych, 2004) primer pair. Protocols and primer pairs are listed in Table S2. PCR products were purified using ExoSap-IT (Thermo Fisher Scientific, Waltham, MA, USA), prepared for Sanger sequencing (BigDye v3.1, using the same primers as in amplification), and sequenced at the University of Bergen inhouse sequencing lab. Chromatogram quality, contigs and manual curation of sequences were done in Geneious 6.1. BLASTN searches were done to confirm that sequence closest relatives were as expected given specimen identity.

### Mock bulk community extraction

Three mock bulk community samples were created using whole specimens from Troll (four grab samples 324–338 m, 78 morphotaxa, 271 specimens), Duva (two grab samples, 350–363 m, 61 morphotaxa, 210 specimens) and Huldra (two grab samples, 121 m, 63 morphotaxa, 256 specimens) after morphological identification. The mock bulk samples were created mimicking a typical grab sample composition by including more specimens from abundant taxa in order to assess the performance of the metabarcoding data on realistic sample compositions.

The mock bulk samples were homogenized for 2 × 30 s using a Qiagen TissueRuptor II (5,000–35,000 rpm) (Qiagen, Hilden, Germany). Five replicate subsamples were retrieved from each sample (15 total) and dried: dry weight 6–8 mg (Troll), 10–12 mg (Duva), and 3–5 mg (Huldra). ATL buffer was added to the subsamples, and they were lysed with proteinase K overnight. Subsequent DNA extraction was done using the Qiagen Blood and Tissue Kit with the QIAsymphony extraction robot (Qiagen).

### HTS sequencing

The 15 mock bulk community sample extracts were amplified with the KAPA3G Plant Kit (Kapa Biosystems) using the modified “Leray” mlCOIintF-XT forward (Wangensteen et al., 2018) and jgHCO2198 reverse (Geller et al., 2013) primer pair for COI, and the SSU_F04 mod (Lanzén et al., 2021) and SSU_R22mod (Sinniger et al., 2016) primer pair for 18S rDNA (Table S2). Twelve random bases were added to primers to aid amplicon sequencing. Library prep was done using the Illumina dual index TruSeq i5/i7 barcode library with equimolar amplicon concentrations. Library products were pooled equimolarly and sent for sequencing using Illumina MiSeq v3 300 bp chemistry at the Norwegian Sequencing Centre (University of Oslo, Norway).

### Bioinformatic processing and data visualization

FastQC v0.11.8 (Andrews, 2010) was used to check the quality of raw FASTQ files. Primer removal was done using Cutadapt v1.18 (Martin, 2011). Filtering, error correction, dereplication, ASV inference, read merging, singleton removal and chimera detection were done using DADA2 (Callahan et al., 2016) (maxEE 2 forward and reverse; truncQ = 2). Sequences outside the expected size range of 313 bp (COI) or 330–450 (18S rDNA) were discarded.

To account for intra-specific genetic variation, DADA2 COI ASVs were clustered into OTUs with SWARM v2.2.2 (Mahé et al., 2015). Initial tests comparing the reduction in the number of OTUs at different SWARM *d* values found a levelling off at *d* = 3 − 5, so a *d* value of 5 was used. This choice was validated by cross-checking that the number of unique assigned species was not lower in the clustered COI data than in test assignments using the unclustered COI ASV data. While several studies have used a *d* = 13 clustering parameter for COI (Brandt et al., 2021; Turon et al., 2020), initial tests found a reduction in number of taxonomically assigned species of around 5% for this setting, and so we choose a less stringent *d* value for COI here. No clustering was done for the 18S rDNA dataset given the more conserved nature of this marker relative to COI. An abundance threshold of 2 × 10^−4^ of each total dataset reads was used to trim the tail of rare OTUs/ASVs from both COI and 18S rDNA datasets. Filtering for cross-contamination was done using R, removing OTUs occurring at very low abundance (<1%) compared to all samples average, analogous to UNCROSS (Edgar, 2016).

Taxonomic assignment was done using CREST4.3.6 (Lanzén et al., 2012) with the MIDORI248 database for COI (Leray, Knowlton & Machida, 2022) (97% species; 95% genus cutoff) and SilvaMod v138 PR2 version 2 database (a taxonomically curated version of the SILVA 138 database with additional PR2 sequences for use with the CREST4 classifier) for 18S rDNA. Barcoded sequences from this study were added manually to these databases to gauge the effectiveness of the targeted barcoding for taxonomic assignment. For COI BOLDigger 2 (Buchner & Leese, 2020) (97% species; 95% genus cutoff) was used as a second classifier with the Barcode of Life Data Systems (BOLD) (Ratnasingham & Hebert, 2007) to gauge relative MIDORI and BOLD database coverage, and to obtain maximum COI assignment coverage by combining both assignment datasets. Finally, some manual COI species assignment was done based on phylogenetic clusters and assessment of BOLDigger 2 results. Taxonomic filtering of non-target taxa included removing all sequences that could not be assigned to phyla found in the morphological dataset, to remove prokaryotes, single-cell eukaryotes, parasites, epizoans, gut content and traces from other taxa. While not part of the general analysis here, non-target OTUs/ASVs are briefly reported in Tables S10–S11. The data was visualized in R v4.3.1 (R Core Team, 2020) using the ggplot2 (Wickham, 2016) and eulerr (Larsson, 2022) packages.

## Results

### Morphological taxonomy and barcoding

Morphological identification of specimens from all stations in the dataset registered 257 morphotaxa for the total number of stations (199 at species rank), with 216 morphotaxa from the North Sea areas (Duva, 75; Huldra, 79; Troll, 113; Fram 89 taxa) and 108 morphotaxa from the Southern Barents Sea (Snøhvit, 48; Goliat, 44; Spissa, 51; Jacob Castberg, 36 taxa). Species overlap between these two areas was 25.7%. All except one taxon (the brachiopod *Macandrevia cranium*) belonged to the phyla Annelida, Mollusca, or Arthropoda (Table S3).

From species lacking COI, 18S rDNA or both markers in NCBI GenBank, between 1–6 specimens, depending on specimen availability, were selected for barcoding. This covered 95 specimens from 45 species. From these 95 specimens, COI sequences were obtained for 47 (49.5%), partial 18S rDNA sequences for 64 (67.4%) and partial 28S rDNA sequences for 50 (52.6%), representing 25, 35, and 24 species respectively (Table S4). The new COI and 18S rDNA sequences were added to the MIDORI 248 and SilvaMod v138 PR2 databases to produce custom versions of these databases used for taxonomic assignment of the mock bulk community samples in this study.

While part of the barcoding and taxonomy effort, five of the areas (Fram, Snøhvit, Goliat, Spissa, and Jacob Castberg) and the generated 28S sequences were not part of the subsequent mock bulk community metabarcoding study here.

### Mock bulk community sample datasets

Three of the eight areas in the full dataset (Duva, Huldra, and Troll) were selected to create mock bulk community samples to compare morphological and metabarcoding data composition and taxonomic assignment: these samples contained 737 specimens from 152 morphotaxa (536 specimens identified to species rank). The Duva sample, pooled from two sampling stations, contained 210 specimens (61 morphotaxa); the Huldra sample, from two sampling stations, 256 specimens (63 morphotaxa); and the Troll sample, from four sampling stations, 271 specimens (78 morphotaxa) (Table S5).

The COI mock bulk community dataset, five replicates from each of the three areas, comprised 2,593,106 raw reads, with 158,977–187,797 reads for individual replicates. Primer removal and DADA2 processing, length filtering, and chimera removal reduced the total read number to 2,131,360 (Table S6). Cross-contamination filtering had only a very modest effect reducing read number by nine reads to 2,131,351. DADA2 identified 482 ASVs in the COI dataset. SWARM clustering (*d* = 5) and abundance filtering reduced this number to 321 and 266 OTUs respectively. Following MIDORI and BOLD taxonomic assignment only phyla that were found in the morphological dataset were retained to allow comparison of morphological and COI data (Arthropoda, Annelida, and Mollusca) comprising 2,006,490 reads in 179 OTUs. Final COI numbers for each area were, 671,959 reads in 58 OTUs for Duva, 716,241 reads in 53 OTUs for Huldra, and 604,862 reads in 65 OTUs for Troll.

The 18S V1–V2 rDNA dataset comprised 2,270,114 reads, with 122,247–168,813 reads for individual replicates. Primer removal and DADA2 processing, length filtering, and chimera removal reduced the total read number to 1,567,937 (Table S6). DADA2 identified 299 ASVs in the 18S rDNA dataset. Abundance filtering reduced this number to 274. Cross-contamination filtering did not reduce the number of reads in the dataset. Following taxonomic assignment and removal of non-target taxa, 1,495,319 reads in 218 ASVs remained. Final 18S rDNA numbers for each area were 501,920 reads in 91 ASVs for Duva, 458,787 reads in 67 ASVs for Huldra, and 534,612 reads in 96 ASVs for Troll. The number of unique taxa in the target phyla (*i.e.,* the four phyla identified in the morphological dataset) found in the morphological and metabarcoding data is given in Table 1 both for the total dataset, and shown separately for the Duva, Huldra, and Troll mock bulk samples, and by phylum (Annelida, Arthropoda, Mollusca, Brachiopoda).

**Table 1:** The number of taxa in the morphological dataset, and the number of target-phylum OTUs in the COI (SWARM2, d = 5) and ASVs in the 18S rDNA (unclustered) datasets. The data is shown both as total and separately for each of the three areas, and for all phyla and individually for the four macrofaunal phyla identified in the morphological dataset (nontarget metazoans in metabarcoding data are listed in Tables S10–S11).

	**Total**	**Annelida**	**Arthropoda**	**Mollusca**	**Brachiopoda**
**Morphology**					
Total	152	83	35	33	1
Duva	61	35	12	13	1
Huldra	63	36	15	11	1
Troll B	78	47	12	19	0
**COI**					
Total	179	85	62	31	0
Duva	80	25	41	14	0
Huldra	57	34	15	8	0
Troll B	74	45	12	16	0
**18S**					
Total	218	151	28	38	1
Duva	91	62	13	15	1
Huldra	67	47	10	9	1
Troll B	96	65	9	22	0

Non-target metazoans in the COI data, that is metazoans not in the morphological data and thus not part of the four phyla in Table 1, included 20 OTUs that could only be identified as Metazoa, and six OTUs at phylum rank or lower, including cnidarians (*Neoturris pileata*, *Metridium* sp.), unidentified Chordata, Holothuroidea, and two nemerteans. Further, 35 OTUs representing 75,990 reads did not produce a hit in either MIDORI or BOLD. Non-target 18S rDNA metazoans included eleven ASVs only identified as Metazoa, and nine ASVs at phylum rank and lower, including anthozoans, the hydrozoan order Anthoathecata, two bryozoans, the nematode order Araeolaimida, the platyhelminth *Gyrocotyle urna*, and the orthonectid parasite *Intoshia linei* (Tables S10–S11).

### Phylum-rank data composition

**Figure 2:**
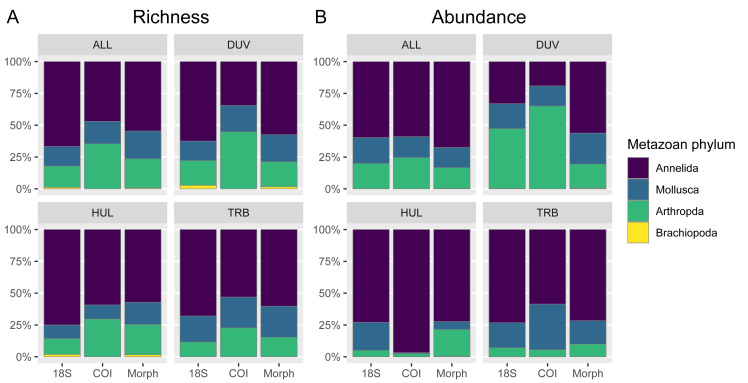
Comparison of relative richness (unique taxa/ASVs/OTUs count) and relative abundance in the morphological and metabarcoding datasets across areas and phyla. (A) The relative proportion of unique taxa (richness), and (B) the relative abundance (*i.e.,* adding specimen and read counts) belonging to the phyla Annelida, Arthropoda, Mollusca, and Brachiopoda in the morphological, COI, and 18S rDNA datasets, shown for the whole dataset, Duva (DUV), Huldra (HUL), and Troll B (TRB).

Taxa from the three major phyla in the morphological dataset—Annelida, Arthropoda, and Mollusca—were all represented in both metabarcoding datasets. The proportional richness of annelid taxa was slightly lower in the COI data, and higher in the 18S rDNA data. Conversely, the proportion of arthropod taxa was slightly higher in the COI dataset due to a high number of arthropod taxa from the Duva sample. Mollusca proportional richness was approximately even between datasets. Finally, Brachiopoda (*Macandrevia cranium*) was missing from the COI data (Fig. 2A).

Adding relative abundances, the proportion of annelid sequence reads was slightly higher than annelid richness in the morphological and COI datasets, while there was a slight decrease in the 18S rDNA dataset. In the morphological data, annelids constituted the most abundant phylum in all three areas. The same pattern was mostly reflected in the metabarcoding data: annelids comprised the most sequence reads in all cases except the Duva COI and 18S rDNA data: for COI, the Duva data was almost completely dominated by the isopod *Natatolana borealis*, which was barely present in the morphological dataset (*n* = 2); in the 18S rDNA data, Duva data showed a much higher proportion of calanoid abundance relative to the morphological and COI datasets. Thus, for both metabarcoding markers, abundance discrepancies were the result of specific abundant OTUs/ASVs (Fig. 2B).

### Taxonomic assignment performance and lower-rank data composition

**Figure 3:**
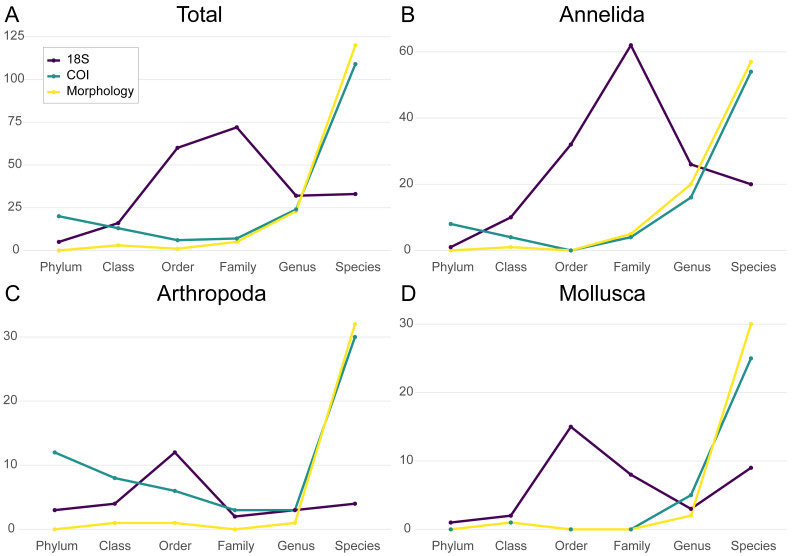
The number of taxa assigned to different taxonomic ranks in the morphological, COI and 18S datasets. Numbers are shown for (A) the total dataset, (B) Annelida, (C) Arthropoda, and (D) Mollusca. Yellow indicates morphological data, green COI, and dark blue 18S rDNA.

Taxonomic identification of morphological specimens classified 120 taxa to species rank, 23 to genus rank, five to family rank, and four to higher taxonomic rank. Of COI OTUs, 66 could be assigned to species rank (58 unique species) using the MIDORI LCA assignment. BOLDigger 2 with manual assessment assigned 110 OTUs to species rank (88 unique species). Forty-five species were common to both MIDORI and BOLD assignments. In 14 cases there was a species identity mismatch between BOLD and MIDORI assignments, and the BOLD assignment was chosen in the final joint assignment dataset, which comprised 118 species-rank (100 unique), 40 genus rank, three family rank, and 18 higher rank OTU assignments (Table S7). In the 18S rDNA dataset, 33 ASVs were resolved to species rank, 32 to genus rank, 72 to family rank, and 81 to higher taxonomic rank (Fig. 3).

The annelid morphological dataset contained 83 taxa spread over a diverse range of polychaete families, two sipunculids, and one oligochaete, 57 of which were identified to species rank (68.7%), and 77 (92.8%) to at least genus rank. The annelid COI dataset included 87 OTUs of which 66 (75.6%) were assigned to species rank (53 unique species) and 78 (89.7%) to at least genus rank. Finally annelid 18S rDNA data comprised 151 unclustered ASVs of which 20 (13.2%) were assigned to species rank (18 unique species) and 48 (31.8%) to at least genus rank (Fig. 3B). Annelid taxon morphological dataset abundance (497 specimens in total) was spread relatively evenly over a wide range of species. In contrast COI read abundance was dominated by two OTUs, *Pista bansei* (55.6%), and *Paradiopatra quadricuspis* (23.1%), with an average of 0.25% abundance for other annelid OTUs. The former OTU is part of the *P. bansei* species complex also found in the morphological dataset (52 of 497 total annelid specimens). In the 18S rDNA data, an order-rank terebellid ASV (potentially *Pista* as well) amounted to 20% of total read number, but 18S rDNA abundances were otherwise somewhat less lopsided than in the COI data (Tables S5, S7–S8).

The arthropod dataset contained 35 morphotaxa, 33 of which were identified to species rank (94.3%). This included 23 amphipod species, five cumaceans, four isopods, and two tanaidaceans in addition to calanoids reported as a single higher-level taxon (Table S5). With 61 total OTUs, the arthropod COI dataset had higher richness than the morphological data, partly due to calanoids not being aggregated together as in the morphological dataset. Of these OTUs, 27 (44.3%) were assigned to species rank (21 unique species), and 50 (82.0%) to at least genus rank. The discrepancy between COI genus and species rank assignment was due to several multi-OTU assignments in the COI data, including the amphipod genera *Ampelisca*, *Haploops*, and *Tmetonyx*, which mostly precluded species-level identification in these cases (Table S7). The COI data included all orders in the morphological data (Amphipoda, Isopoda, Tanaidacea, Calanoida, and Cumacea). With 28 ASVs, of which only four (14.3%) could be assigned to species level and seven (25.0%) to at least genus level, the 18S rDNA dataset contained the fewest arthropod taxa among the three datasets (Fig. 3C).

Arthropod taxon abundance in the morphological dataset (121 specimens in total) showed a low (1–7) number of specimens for all taxa except the higher-rank Calanoida group containing calanoids not identified to a lower taxonomic rank (*n* = 29) and the amphipod *Unciola planipes* (*n* = 20 at Huldra exclusively). Relative to morphological count, metabarcoding arthropod read abundance proved highly biased in both COI and 18S rDNA data. In the case of COI, the isopod *Natatolana borealis* (only present at Duva as two specimens in the morphological data) represented 68.8% of all COI reads. Twelve of the 28 18S rDNA ASVs were calanoids, and of these, two unknown calanoid ASVs represented 96.5% of all 18S rDNA arthropod read abundance (Tables S7–S8).

The morphological mollusk dataset contained 33 taxa, with all but one (Caudofoveata) identified to species level. The mollusk COI dataset contained 31 OTUs of which 25 (80.6%) were assigned to species rank (24 unique species) and 30 (96.8%) to at least genus rank. The protobranch bivalve genus *Yoldiella*, represented by six OTUs, was mostly unresolved at species level. *Yoldiella* COI species assignments did not correspond with morphological taxonomy or 18S rDNA assignment, highlighting the taxonomic complexity of this genus (Killeen & Turner, 2009) (Tables S5, S7–S8). Mollusk 18S rDNA data comprised 38 unclustered ASVs of which 9 (23.7%) were assigned to species rank (7 unique species) and 12 (31.6%) to at least genus rank (Fig. 3D).

For taxa identified as mollusks, all classes in the morphological dataset (Bivalvia, Gastropoda, Scaphopoda, Caudofoveata, Solenogastres, Polyplacophora) were well represented in terms of number of taxa in both COI and 18S rDNA metabarcoding data. COI dataset abundance favored taxa from several classes, including the bivalves *Abra longicallus* and *A. nitida*, the scaphopod *Entalina tetragona*, and the caudofoveate *Scutopus ventrolineatus*, and showed no systematic high-rank taxonomic bias. Similarly, for the 18S rDNA data, while dominated by a single unknown bivalve, *E. tetragona*, *Leptochiton asellus*, and *S. ventrolineatus* were also well represented (Tables S7–S8).

Fourteen of the species barcoded for this study were present in the mock bulk community samples from, Duva, Huldra, and Troll. These new barcodes increased the taxonomic resolution of six OTUs in the COI metabarcoding dataset, and five ASVs in the 18S rDNA dataset, to species or genus rank. This represented, on average, a resolution increase of 4–5 taxonomic ranks, typically from phylum or order rank up to species—or in one case genus—rank (Table S9).

### Morphological and metabarcoding taxonomy correspondence

**Figure 4:**
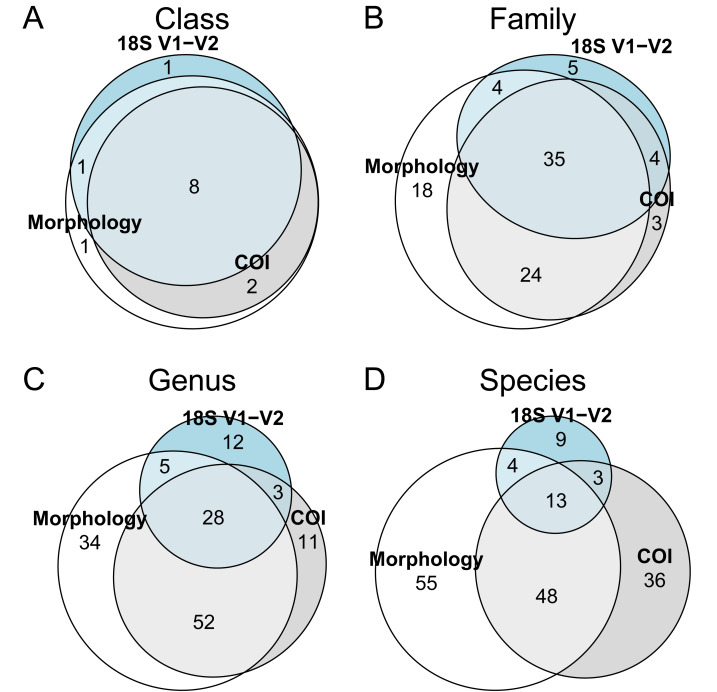
Euler diagrams showing the degree of overlap in identified taxa at (A) class, (B) family, (C) genus, and (D) species taxonomic rank between the morphological, COI and 18S rDNA datasets. Numbers signify the number of taxa at a particular taxonomic rank that were recovered in all three, two or only one of the three datasets. Taxon names were manually checked to identify discrepancies in database names and classification.

**Figure 5:**
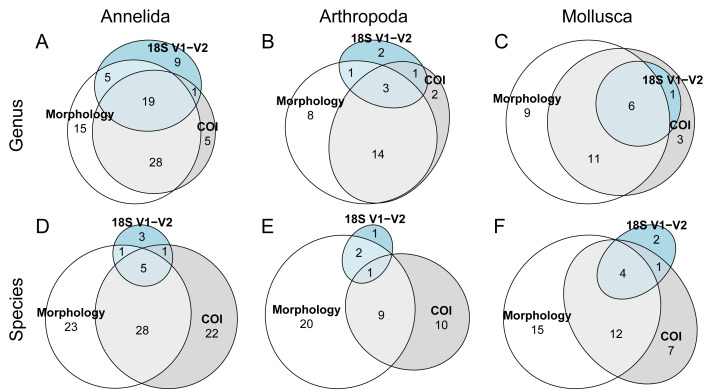
Euler diagrams showing the degree of overlap between the morphological, COI and 18S rDNA datasets in identified taxa at (A–C) genus, and (D–F) species rank. Data is shown separately for (A, D) Annelida, (B, E) Arthropoda, and (C, F) Mollusca.

The extent of taxon overlaps between the morphological, COI and 18S rDNA datasets is shown in Fig. 4, showing a decrease in overlap at lower taxonomic rank (Figs. 4A–4D). The number of unique taxa at lower taxonomic rank is somewhat higher in the morphological dataset, though there is also a significant number of species only reported in the COI data, likely partly due to incongruences in species-rank assignment for these two methods. The 18S rDNA data has the smallest number of identified taxa at lower taxonomic rank, but assigned genera and species included some taxa that were not present in the COI data (Fig. 4D). Looking at data separately for the three phyla at genus and species level, overall patterns are roughly similar: in all three phyla, species-rank identification shows a divergence between morphological and COI assignment, with 18S rDNA constituting a minor addition to the overall number of reported species given the relative lack of species-level resolution (Figs. 5A–5E). Due to unresolved amphipod OTU complexes found in the COI data, species resolution for COI arthropod data is particularly low (Fig. 5E).

## Discussion

### Major phyla were well represented in metabarcoding data

Metabarcoding datasets invariably includes biases due to primer site mismatches, meaning a certain fraction of taxa can be expected to remain undetected (Albaina, Gari’c & Yebra, 2024). The metabarcoding sample type—eDNA (sediment, water) *vs.* bulk sample—also have a critical influence on the typical data profile: COI data from environmental samples typically have a much higher number of unique sequences representing low-abundance unassignable reads, and conversely typically recovers fewer known macrofaunal species and less overlap between morphological and metabarcoding data (Hestetun, Lanzén & Dahlgren, 2021; Good et al., 2022; Pawlowski et al., 2022; Willassen et al., 2022).

For bulk community samples, previous studies that have investigated metazoan macrofaunal datasets using COI and 18S rDNA metabarcoding data have found various levels of taxon coverage (Andújar et al., 2018; Lejzerowicz et al., 2015; Leray et al., 2013). In a comparative study of COI primer pairs, Derycke et al. (2021) found that standard Leray primers (mlCOIintF) recovered more assignable ASVs than other COI primer alternatives. While that study failed to produce results using the modified mlCOIintF-XT Leray forward primer used in this study, other studies have used this primer variant with good results (Hestetun, Lanzén & Dahlgren, 2021; Lanzén et al., 2021; Turon et al., 2020) and the original article (Wangensteen et al., 2018) claims higher *in-silico* metazoan coverage than the unmodified Leray marker. Further COI *in-silico* primer set comparisons by Ren, Zhou & Zhang (2025) confirmed and recommended the mlCOIintF-XT/jgHCO2198 primer set used here to recover broadest possible metazoan amplification (though that study also found bias towards common macrofaunal phyla compared to others).

Some studies have shown that Leray COI recovery of annelids is relatively lacking. For instance, Derycke et al. (2021) found that eleven out of 19 morphospecies not recovered in the metabarcoding data were polychaetes. Lejzerowicz et al. (2015) found that while annelid abundance was high, richness at family rank was lower in the COI data (*n* = 12) than in the corresponding morphological dataset (*n* = 26). On the other hand, annelids formed the majority of the data in both morphological and Leray COI datasets in Steyaert et al. (2020), implying that a good number of annelid clades do amplify using this COI primer pair, and Ren, Zhou & Zhang (2025) reported high *in-silico* coverage for annelids using the mlCOIintF-XT Leray forward primer.

We could not see any clear bias against polychaetes in the COI data here: Proportional COI richness was somewhat lower for Annelida than in morphological and 18S rDNA datasets, especially at Huldra (Fig. 2), but this was due to a larger number of arthropod OTUs rather than a low number of annelid OTUs (85 annelid OTUs *vs.* 83 annelid taxa in the morphological dataset; 62 arthropod COI OTUs *vs.* 35 arthropod morphotaxa) (Table 1). Further, 29 out of 35 annelid families found in the morphological dataset were recovered in the COI data (Table S5, S7). The high annelid sequence coverage in the study area thus allowed a good representation of polychaete clades in the COI data. On the other hand, the two oligochaete specimens and the two sipunculid *Onchnesoma* species reported from the morphological data were not recovered in the COI data (rather the sipunculid *Aspidosiphon muelleri*).

In the case of Arthropoda, several prior studies (Casey et al., 2021; Cowart et al., 2015; Ransome et al., 2017) have shown a high number of arthropods assigned species rank in the COI data. Species-rank resolution was somewhat lower here, but this was mostly due to the unresolved amphipod species complexes in the data (Fig. 3). Finally for mollusks, low representation has also been reported for Leray COI data (Lejzerowicz et al., 2015). For instance, Cahill et al. (2018), found that mollusks were severely underrepresented in their data, though their presented data does not show whether this represented low amplification success for a few species of abundant bivalves, or whether this under-performance was consistent over a wider selection of mollusk species. In contrast, we observed high recovery for mollusks, where the COI marker was able to reproduce a variety of taxa from all five mollusk classes included in the morphological dataset. Noth arthropods and mollusks were also well-represented in the *in-silico* analysis of Ren, Zhou & Zhang (2025).

In sum, the data in this study showed high COI performance for all three major phyla using the Leray mlCOIintF-XT-jgHCO2198 primer pair. The single identified species not part of the three major phyla, the brachiopod *Macandrevia cranium*, was missing from the COI dataset, a finding similar to Grey et al. (2018). Given that this species is present in the MIDORI database, this implies that this species is not readily recovered by the COI primer combination used here, and low affinity to this primer combination for Brachiopoda was also reported by Ren, Zhou & Zhang (2025).

Several studies, *e.g.*, Cowart et al. (2015), have reported lower performance for 18S rDNA assignment, and this marker has previously been described as lacking needed species-rank resolution for a complete census of marine life (Leray & Knowlton, 2016). In the case of meiofauna, Tang et al. (2012) reported that 18S rDNA under-represented morphological biodiversity in their data by a factor of 0.4. For this reason, we chose to not cluster 18S rDNA data in this study. We did see a significantly lower level of assignment success for 18S rDNA relative to that of COI. The major reason for this discrepancy was the relative lack of taxon coverage in the SILVA database compared to COI coverage in BOLD and MIDORI databases, but the 18S rDNA marker here did recover 30 separate species-rank assignments. To the extent that related species have similar 18S rDNA sequence identity (*i.e.,* lack of species-rank resolution), we do note that LCA assignment could actually produce fewer species-rank assignments in future cases with higher taxonomic database coverage, where the query sequence is equally similar to more than one species deposited in the assignment database. In such cases, the LCA algorithm would default to a higher rank.

The 18S rDNA dataset, while providing much fewer genus and species rank assignments, produced a higher proportion of intermediate-rank (family, order) identifications. This compared favorably to the COI data, where OTUs with no low-rank match could be identified to *e.g.*, only kingdom or phylum rank, consistent with previous studies (Wangensteen et al., 2018) (Fig. 3). This is likely due to the stem-loop resolution pattern of the ribosomal 18S rDNA sequence compared to the overall higher divergence rate (and potential oversaturation) in the protein-coding COI gene.

We did not see any major 18S rDNA data biases in annelid or mollusk data. The primer pair used to amplify the 18S rDNA V1–V2 partition also recovers numerous annelids and was found to have comparable results to morphology in species detection (though not abundance) by Klunder et al. (2022). Arthropods were poorly represented in the 18S rDNA data, however: here a number of calanoid ASVs formed the vast majority of the dataset, and the diversity of amphipods in the morphological data was almost entirely absent (Table S8).

An additional challenge in taxonomic classification in 18S rDNA data is that the SILVA database does not include the full amount of 18S rDNA sequences from *e.g.*, NCBI GenBank due to time from GenBank publication and inclusion into the database (“curation”). It is thus possible that custom databases, or recent alternatives such as the EUKARYOME database (Tedersoo et al., 2024), could represent attractive alternatives, though this is beyond the scope of the current study. While overall the performance of 18S rDNA in taxonomic assignment here was low relative to COI, we note that eight genera and species were shared only between the morphological and 18S rDNA datasets (*i.e.,* not with COI). Using multiple markers thus allowed improved recovery of the benthic community, and given increased taxonomic database coverage in the future, multi-marker approaches should be seriously considered for any given metabarcoding study to compensate for non-amplifications due of species to inherent marker biases in the COI data (Alberdi et al., 2018; Van der Loos & Nijland, 2021).

### Abundance biases were due to disparate taxa

Morphology and metabarcoding dataset congruence decreased somewhat when considering the data separately for the three studied areas, in particular abundance data. Examples included an over-representation of annelids in the COI data, and under-representation of arthropods at Huldra in both COI and 18S rDNA data; in contrast there was an over-representation of arthropods and under-representation of annelids in both metabarcoding datasets at Duva (Fig. 2).

It has been repeatedly shown that PCR-based data often show extreme abundance biases in metabarcoding datasets, severely limiting the application of metabarcoding abundance data. For instance, Bijleveld et al. (2018) found poor correlation between morphological biomass or abundance and metabarcoding occupancy data. Extremely abundant OTUs are common in metazoan COI datasets (*e.g.*, Hestetun, Lanzén & Dahlgren, 2021). Similar biases in the metabarcoding data in this study were thus unsurprising.

We wanted to see if we could discern any high-rank taxonomic pattern to such bias, but our findings instead showed that for both metabarcoding datasets, extreme abundance biases were due to single OTUs/ASVs from different taxonomic groups rather than specific clades: the COI and 18S rDNA datasets both featured extremely abundant single annelids, and mollusks from several of the mollusk classes. Further, the most extreme examples of single-taxon bias in the metabarcoding data were arthropods: the isopod *Natatolana borealis* (COI) and an unresolved calanoid (18S rDNA). Significantly, specific taxa mostly did not overlap between the COI and 18S rDNA datasets, supporting the conclusions of Kelly et al. (2017) that this bias is driven primarily by primer and template DNA interactions rather than relative abundance of DNA in the source extracts. Given these findings, our interpretation here is that such optimal primer-template interactions are not systematically restricted to certain clades, however, but are found in lower taxa across several phyla for the two sets of primer pairs used in this study.

### Datasets diverged at species rank—even with high sequence coverage

Taxonomic coverage—the fraction of species with relevant sequence information in online databases—is critical to allow assessment of congruence between morphological and macrofaunal metabarcode data. In this regard, the North Sea and adjacent areas may represent a best-case scenario in terms of previous and ongoing efforts to populate these databases with faunal sequences through taxonomic research conducted at institutions in bordering countries including regional Barcode of Life initiatives (Hestetun et al., 2020).

Due to this high coverage, it proved possible to assign a relatively high proportion of COI OTUs to low taxonomic rank using the combined Crest4/MIDORI and BOLDigger2/BOLD method to maximize database coverage here, in all 100 unique species, compared to 127 unique taxa in the morphological dataset. This is an improvement compared to previous studies (Steyaert et al., 2020; Wangensteen et al., 2018) where species-rank assignments performed less well due to lower taxonomic coverage.

An interesting aspect of the COI species assignment here proved to be the divergence in taxonomic assignment between the morphological data and the COI dataset at species rank: while 52 species were shared across these datasets, there were 66 morphospecies and 39 unique COI OTU assignments in their respective non-shared portion of the data (Fig. 4D). A quick look at the assignments for both methods (Tables S5, S7) revealed that many of these assignments agreed on genus, but disagreed on species identity: the COI OTUs thus represented close but alternative assignments of taxa found in the morphological dataset rather than *e.g.*, parasites, fragments *etc.* not part of this data.

The exact results of this type of comparison are subject to fine tuning of the relative species- and genus-level thresholds for OTU assignments, yet species-level barcode gaps vary between invertebrate clades so that any threshold represents a tradeoff. More interesting is that such discrepancies shine a light on genotype diversity within morphospecies including species complexes. Many morphological taxa are known or suspected to encompass considerable variation based on observations on morphological variation, geographical extent or depth range. Incongruences in morphology and metabarcoding low-rank assignments thus present excellent opportunities to identify potential species complexes for further taxonomic and barcoding studies.

On the other hand, some of the species OTU clusters here were likely the result of technical artifacts during sample processing, such as the proliferation of *Ampelisca* and *Haploops* OTUs at Duva (11 and 14 respectively) (Table S7), a higher number than the actual specimens included in the community samples (three and seven) (Table S5). The fact that these clusters persisted even after SWARM clustering shows that both richness and genotype variation assessments for metabarcoding datasets need to be treated with caution and examined for errors that inflate apparent diversity: not all ASVs or OTUs represent true genotype variation but may represent sequencing artifacts not removed even by state-of-the-art bioinformatic pipelines.

### Targeted barcoding, specificity, and non-target organisms

As part of this study, we added additional barcodes to existing database coverage to gauge the effectiveness of complementary targeted barcoding. For the barcoding effort, we identified specimens from a larger 32 station dataset from both the Northern North and Southern Barents Seas and specifically looked for species where we found complete or partial gaps in sequence coverage, *i.e.,* a targeted approach to increase regional barcode coverage. Here, we wanted to maximize the utility of the barcodes for future studies by extending the barcodes beyond the sequences used for metabarcoding here (*i.e.,* full Folmer COI, 28S rDNA and longer 18S rDNA sequences). For overlapping parts of the COI and 18S rDNA to the metabarcoding markers used here, these barcodes increased assignment accuracy for eleven OTUs/ASVs from each metabarcoding dataset. Comparing taxonomic assignment of the metabarcoding data in this study prior to and after adding sequences from the newly barcoded species here, overall rank of assignment for these OTUs typically increased by 4–6 taxonomic ranks, *i.e.,* from kingdom, phylum, or class rank to species rank. This is an interesting result as it shows that even when sequences are present in online databases from related species, this might be of limited help in species assignment due to sequence saturation. Our findings thus show that closely matching sequences are necessary in order to provide accurate low-rank taxonomic assignment. While this has been noted for COI previously (Casey et al., 2021; Ransome et al., 2017), results here reinforce this view for 18S rDNA as well. Our findings thus show that unassignable or low-rank OTUs/ASVs might easily represent species part of the morphological dataset.

On the other hand, while the metabarcoding data in this study included only taxa at least assigned to a phylum identified in the morphological data, as often noted (Derycke et al., 2021; Lejzerowicz et al., 2015; Lejzerowicz et al., 2021; Ransome et al., 2017), OTUs/ASVs assigned to only phylum rank also likely include metazoans not examined as part of standard >1 mm macrofaunal studies, such as small, cryptic, or pelagic species that are not well-described morphologically, or even fragments, larvae, parasites, gut content, or extracellular DNA. Indeed, one of the often-mentioned advantages of metazoan metabarcoding is the ability to readily identify taxonomic groups which have traditionally not been included or only identified to a high rank in traditional morphological softbottom surveys such as sponges, cnidarians or meiofaunal phyla (Lejzerowicz et al., 2015). Conversely, some of the identified species in the morphological dataset are probably not represented in the metabarcoding data at all and may prove difficult to sequence with the markers used here. Given that the forward “Folmer” primer or a variant thereof (*e.g.*, Geller et al., 2013) typically used in COI barcoding is in a different position than the “Leray” forward primer used for metabarcoding, even species with taxonomic database coverage might not show up in corresponding metabarcoding datasets due to poor primer fit, as shown for instance in Derycke et al. (2021). Thus, even with increased barcoding effort, many OTUs will likely remain assignable to high taxonomic rank only for the foreseeable future.

In this study, metazoan phyla not in the morphological dataset were detected in both COI and 18S rDNA metabarcoding data, including a small number of bryozoans, chordates cnidarians, echinoderms, nemerteans, nematodes, orthonectids, and platyhelminths, though they were not included in subsequent analysis here. The most abundant OTUs/ASVs outside the scope of the target phyla in both COI and 18S rDNA datasets were those that were not identifiable at all, or only assigned as unknown metazoans, however, representing 75,990 reads in 35 OTUs for COI and 52,296 reads in 11 ASVs for 18S rDNA (Tables S10–S11). While the relative proportion of these reads that represent species present in the morphological species inventory *versus* parasites, meiofauna, or fragments is unknown, this shows that there is still potential for higher taxonomic coverage in the datasets in this study.

While quantitative biodiversity estimates remain problematic for current PCR-based metabarcoding, species inventories and checklists could represent an attractive and cost-effective complement to current methodology in characterizing softbottom habitats and the detection of keystone and invasive species. Given the huge effort already being undertaken in collecting, sorting, and identifying softbottom macrofauna as part of current research and environmental baseline and monitoring campaigns, complementary targeted barcoding and metabarcoding studies could readily provide major extra utility for little extra effort. To the extent that new habitats become relevant for increased monitoring, such as the deep sea, targeted barcoding and metabarcoding studies should be considered part of the study design. This implies increased co-operation and involvement between current monitoring stakeholders and consultants with the scientific taxonomic, barcoding and metabarcoding establishment.

## Conclusions

Use of bulk community metabarcoding data in benthic metazoan biodiversity surveys is dependent both on the capacity to recover a wide range of relevant taxa, and the ability to assign sequences to known species. Using mock bulk samples with a known taxonomic composition, we found generally high taxon recovery in the COI metabarcoding data for annelids, arthropods, and mollusks. Abundance biases were clear but distributed across different taxonomic groups in the dataset. By combining the BOLD and MIDORI databases, we were able to detect a comparable number of species-rank taxa to that of the morphological dataset. Taxonomic assignments showed some divergence at species rank, partly due genotypic variation, showing how metabarcoding could also be used as a tool to aid the resolution of species complexes. The 18S rDNA data recovered fewer taxa, and arthropod data was clearly biased towards copepods but recovered the sole brachiopod and other taxa not found in the COI data, showing that a multi-marker approach helps mitigate primer selection bias. Finally, the results underscored the importance of barcoding initiatives to the quality of subsequent taxonomic assignment.

This study shows the potential of an integrated morphological and metabarcoding approach to mapping and understanding benthic macrofaunal biodiversity: Morphological taxonomy provides detailed species identifications and abundance estimates but may overlook cryptic diversity and taxa that are difficult to distinguish visually. Metabarcoding improves sample processing capacity in presence-absence habitat mapping, detect additional organisms, and can reveal hidden genetic diversity. Key to this integration is the continual improvement of taxonomic sequence coverage in online databases afforded by targeted barcode approaches for markers of interest. We would thus recommend adding COI (and potentially 18S or *e.g.*, 12S rDNA) metabarcoding to complement future morphotaxonomic baseline and environmental surveys on the NCS and increase co-operation between environmental monitoring stakeholders in government and industry, and the taxonomic specialist community. By comparing metabarcoding datasets against morphological species lists in this parallel approach, biases and additional biodiversity can be better known even at low taxonomic rank, providing the necessary context to use the metabarcode data in biodiversity and impact assessment.

##  Supplemental Information

10.7717/peerj.20849/supp-1 Supplemental Information 1 In-depth information on sampling stations, PCR conditions, run statistics, and morphological, COI and 18S taxon/OTU/ASV tables

## Additional Information and Declarations

### Field Study Permissions

The following information was supplied relating to field study approvals (*i.e.*, approving body and any reference numbers): No permit is required under Norwegian legislation for the sampling of non-cephalopod invertebrates that are (1) not from a foreign country, (2) not from restricted species or protected areas, and (3) not subject to scientific experiments. Relevant legislation includes (1) the Norwegian Nature Diversity Act (section 58), (https://www.regjeringen.no/en/documents/nature-diversity-act/id570549/), and (2) the regulation on the use of animals in experiments (https://lovdata.no/dokument/SF/forskrift/2015-06-18-761/).

### Data Availability

The following information was supplied regarding data availability: The barcoded specimen DNA sequences are available at NCBI GenBank: OQ053019 –OQ053065 (COI), OQ071217 –OQ071280 (18S rDNA), and OQ071281 –OQ071330 (28S rDNA).
PRJNA1297304, SAMN50217213 –SAMN50217230, and SRR34726514 –SRR34726549.

## References

[ref-1] Albaina A, Gari’c RG, Yebra L (2024). Know your limits; miniCOI metabarcoding fails with key marine zooplankton taxa. Journal of Plankton Research.

[ref-2] Alberdi A, Aizpurua O, Gilbert MTP, Bohmann K (2018). Scrutinizing key steps for reliable metabarcoding of environmental samples. Methods in Ecology and Evolution.

[ref-3] Andrews S (2010). FastQC-a quality control application for FastQ files. http://www.bioinformatics.babraham.ac.uk/projects/fastqc/.

[ref-4] Andújar C, Arribas P, Yu DW, Vogler AP, Emerson BC (2018). Why the COI barcode should be the community DNA metabarcode for the metazoa. Molecular Ecology.

[ref-5] Aylagas E, Borja Á, Irigoien X, Rodríguez-Ezpeleta N (2016). Benchmarking DNA metabarcoding for biodiversity-based monitoring and assessment. Frontiers in Marine Science.

[ref-6] Aylagas E, Borja Á, Muxika I, Rodríguez-Ezpeleta N (2018). Adapting metabarcoding-based benthic biomonitoring into routine marine ecological status assessment networks. Ecological Indicators.

[ref-7] Bijleveld AI, Compton TJ, Klunder L, Holthuijsen S, Ten Horn J, Koolhaas A, Dekinga A, Van Der Meer J, Van Der Veer HW (2018). Presence-absence of marine macrozoobenthos does not generally predict abundance and biomass. Scientific Reports.

[ref-8] Brandt MI, Trouche B, Quintric L, Günther B, Wincker P, Poulain J, Arnaud-Haond S (2021). Bioinformatic pipelines combining denoising and clustering tools allow for more comprehensive prokaryotic and eukaryotic metabarcoding. Molecular Ecology Resources.

[ref-9] Buchner D, Leese F (2020). BOLDigger –a Python package to identify and organise sequences with the Barcode of Life Data systems. Metabarcoding and Metagenomics.

[ref-10] Cahill AE, Pearman JK, Borja A, Carugati L, Carvalho S, Danovaro R, Dashfield S, David R, Féral JP, Olenin S, Šiaulys A, Somerfield PJ, Trayanova A, Uyarra MC, Chenuil A (2018). A comparative analysis of metabarcoding and morphology-based identification of benthic communities across different regional seas. Ecology and Evolution.

[ref-11] Callahan BJ, McMurdie PJ, Rosen MJ, Han AW, Johnson AJA, Holmes SP (2016). DADA2: high-resolution sample inference from Illumina amplicon data. Nature Methods.

[ref-12] Carr CM, Hardy SM, Brown TM, Macdonald TA, Hebert PDN (2011). A tri-oceanic perspective: DNA barcoding reveals geographic structure and cryptic diversity in Canadian polychaetes. PLOS ONE.

[ref-13] Casey JM, Ransome E, Collins AG, Mahardini A, Kurniasih EM, Sembiring A, Schiettekatte NMD, Cahyani NKD, Wahyu Anggoro A, Moore M, Uehling A, Belcaid M, Barber PH, Geller JB, Meyer CP (2021). DNA metabarcoding marker choice skews perception of marine eukaryotic biodiversity. Environmental DNA.

[ref-14] Clarke LJ, Soubrier J, Weyrich LS, Cooper A (2014). Environmental metabarcodes for insects: in silico PCR reveals potential for taxonomic bias. Molecular Ecology Resources.

[ref-15] Collins RA, Bakker J, Wangensteen OS, Soto AZ, Corrigan L, Sims DW, Genner MJ, Mariani S (2019). Non-specific amplification compromises environmental DNA metabarcoding with COI. Methods in Ecology and Evolution.

[ref-16] Cordier T, Alonso-Sáez L, Apothéloz-Perret-Gentil L, Aylagas E, Bohan DA, Bouchez A, Chariton A, Creer S, Frühe L, Keck F, Keeley N, Laroche O, Leese F, Pochon X, Stoeck T, Pawlowski J, Lanzén A (2021). Ecosystems monitoring powered by environmental genomics: a review of current strategies with an implementation roadmap. Molecular Ecology.

[ref-17] Cowart DA, Pinheiro M, Mouchel O, Maguer M, Grall J, Miné J, Arnaud-Haond S (2015). Metabarcoding is powerful yet still blind: a comparative analysis of morphological and molecular surveys of seagrass communities. PLOS ONE.

[ref-18] Deagle BE, Jarman SN, Coissac E, Pompanon F, Taberlet P (2014). DNA metabarcoding and the cytochrome c oxidase subunit I marker: not a perfect match. Biology Letters.

[ref-19] Deiner K, Bik HM, Mächler E, Seymour M, Lacoursière-Roussel A, Altermatt F, Creer S, Bista I, Lodge DM, de Vere N, Pfrender ME, Bernatchez L (2017). Environmental DNA metabarcoding: transforming how we survey animal and plant communities. Molecular Ecology.

[ref-20] Derycke S, Maes S, Van den Bulcke L, Vanhollebeke J, Wittoeck J, Hillewaert H, Ampe B, Haegeman A, Hostens K, De Backer A (2021). Detection of macrobenthos species with metabarcoding is consistent in bulk DNA but dependent on body size and sclerotization in eDNA from the ethanol preservative. Frontiers in Marine Science.

[ref-21] DNV (2023). https://mod.dnv.com/.

[ref-22] Edgar RC (2016). UNCROSS: filtering of high-frequency cross-talk in 16S amplicon reads. BioRxiv.

[ref-23] Geller J, Meyer C, Parker M, Hawk H (2013). Redesign of PCR primers for mitochondrial cytochrome c oxidase subunit I for marine invertebrates and application in all-taxa biotic surveys. Molecular Ecology Resources.

[ref-24] Gielings R, Fais M, Fontaneto D, Creer S, Costa FO, Renema W, Macher JN (2021). DNA metabarcoding methods for the study of marine benthic meiofauna: a review. Frontiers in Marine Science.

[ref-25] Good E, Holman LE, Pusceddu A, Russo T, Rius M, Iacono Clo (2022). Detection of community-wide impacts of bottom trawl fishing on deep-sea assemblages using environmental DNA metabarcoding. Marine Pollution Bulletin.

[ref-26] Grey EK, Bernatchez L, Cassey P, Deiner K, Deveney M, Howland KL, Lacoursière-Roussel A, Leong SCY, Li Y, Olds B, Pfrender ME, Prowse TAA, Renshaw MA, Lodge DM (2018). Effects of sampling effort on biodiversity patterns estimated from environmental DNA metabarcoding surveys. Scientific Reports.

[ref-27] Hadziavdic K, Lekang K, Lanzen A, Jonassen I, Thompson EM, Troedsson C (2014). Characterization of the 18S rRNA gene for designing universal eukaryote specific primers. PLOS ONE.

[ref-28] He X, Sutherland TF, Abbott CL (2021). Improved efficiency in eDNA metabarcoding of benthic metazoans by sieving sediments prior to DNA extraction. Environmental DNA.

[ref-29] Hestetun JT, Bye-Ingebrigtsen E, Nilsson RH, Glover AG, Johansen P-O, Dahlgren TG (2020). Significant taxon sampling gaps in DNA databases limit the operational use of marine macrofauna metabarcoding. Marine Biodiversity.

[ref-30] Hestetun JT, Lanzén A, Dahlgren TG (2021). Grab what you can—an evaluation of spatial replication to decrease heterogeneity in sediment eDNA metabarcoding. PeerJ.

[ref-31] Jaksch K, Eschner A, Rintelen TV, Haring E (2016). DNA analysis of molluscs from a museum wet collection: a comparison of different extraction methods. BMC Research Notes.

[ref-32] Kelly RP, Closek CJ, O’Donnell JL, Kralj JE, Shelton AO, Samhouri JF (2017). Genetic and manual survey methods yield different and complementary views of an ecosystem. Frontiers in Marine Science.

[ref-33] Kessing B, Croom H, Martin A, McIntosh C, Mcmillan WO, Palumbi S (1989). The simple fool’s guide to PCR.

[ref-34] Killeen I, Turner J (2009). Yoldiella and Portlandia (Bivalvia) from the Faroe-Shetland Channel and Rockall Trough, Northeast Atlantic. Journal of Conchology.

[ref-35] Klunder L, van Bleijswijk JDL, Kleine Schaars L, van der Veer HW, Luttikhuizen PC, Bijleveld AI (2022). Quantification of marine benthic communities with metabarcoding. Molecular Ecology Resources.

[ref-36] Lanzén A, Dahlgren TG, Bagi A, Hestetun JT (2021). Benthic eDNA metabarcoding provides accurate assessments of impact from oil extraction, and ecological insights. Ecological Indicators.

[ref-37] Lanzén A, Jørgensen SL, Huson DH, Gorfer M, Grindhaug SH, Jonassen I, Øvreås L, Urich T (2012). CREST—classification resources for environmental sequence tags. PLOS ONE.

[ref-38] Laroche O, Kersten O, Smith CR, Goetze E (2020). Environmental DNA surveys detect distinct metazoan communities across abyssal plains and seamounts in the western Clarion Clipperton Zone. Molecular Ecology.

[ref-39] Larsson J (2022). https://CRAN.R-project.org/package=eulerr.

[ref-40] Leduc N, Lacoursière-Roussel A, Howland KL, Archambault P, Sevellec M, Normandeau E, Dispas A, Winkler G, McKindsey CW, Simard N, Bernatchez L (2019). Comparing eDNA metabarcoding and species collection for documenting Arctic metazoan biodiversity. Environmental DNA.

[ref-41] Lejzerowicz F, Esling P, Pillet L, Wilding TA, Black KD, Pawlowski J (2015). High-throughput sequencing and morphology perform equally well for benthic monitoring of marine ecosystems. Scientific Reports.

[ref-42] Lejzerowicz F, Gooday AJ, Barrenechea Angeles I, Cordier T, Morard R, Apothéloz-Perret-Gentil L, Lins L, Menot L, Brandt A, Levin LA, Martinez Arbizu P, Smith CR, Pawlowski J (2021). Eukaryotic biodiversity and spatial patterns in the clarion-clipperton zone and other abyssal regions: insights from sediment DNA and RNA metabarcoding. Frontiers in Marine Science.

[ref-43] Leray M, Knowlton N (2016). Censusing marine eukaryotic diversity in the twenty-first century. Philosophical Transactions of the Royal Society B: Biological Sciences.

[ref-44] Leray M, Knowlton N, Machida RJ (2022). MIDORI2: a collection of quality controlled, preformatted, and regularly updated reference databases for taxonomic assignment of eukaryotic mitochondrial sequences. Environmental DNA.

[ref-45] Leray M, Yang JY, Meyer CP, Mills SC, Agudelo N, Ranwez V, Boehm JT, Machida RJ (2013). A new versatile primer set targeting a short fragment of the mitochondrial COI region for metabarcoding metazoan diversity: application for characterizing coral reef fish gut contents. Frontiers in Zoology.

[ref-46] Lobo J, Shokralla S, Costa MH, Hajibabaei M, Costa FO (2017). DNA metabarcoding for high-throughput monitoring of estuarine macrobenthic communities. Scientific Reports.

[ref-47] Mahé F, Rognes T, Quince C, de Vargas C, Dunthorn M (2015). Swarm v2: highly-scalable and high-resolution amplicon clustering. PeerJ.

[ref-48] Mannvik H-P, Wasbotten IH, Andrade H (2020). Overvåkings- og grunnlagundersøkelser i Barentshavet, 2019. Akvaplan-niva AS Rapport. 60711.03.

[ref-49] Martin M (2011). Cutadapt removes adapter sequences from high-throughput sequencing reads. EMBnet.Journal.

[ref-50] Mauffrey F, Cordier T, Apothéloz-Perret-Gentil L, Cermakova K, Merzi T, Delefosse M, Blanc P, Pawlowski J (2021). Benthic monitoring of oil and gas offshore platforms in the North Sea using environmental DNA metabarcoding. Molecular Ecology.

[ref-51] Møskeland T, Brooks L, Ulfsnes A (2020). Offshore miljøovervåking, Region III 2019. DNV-GL Report, 2020-0246.

[ref-52] Murray A, Priest T, Antich A, von Appen WJ, Neuhaus S, Havermans C (2024). Investigating pelagic biodiversity and gelatinous zooplankton communities in the rapidly changing European Arctic: an eDNA metabarcoding survey. Environmental DNA.

[ref-53] Norwegian Environment Agency (2020). M-408. Guidelines for environmental monitoring of petroleum activities on the Norwegian continental shelf, rev. 2020.

[ref-54] Passamaneck YJ, Schander C, Halanych KM (2004). Investigation of molluscan phylogeny using large-subunit and small-subunit nuclear rRNA sequences. Molecular Phylogenetics and Evolution.

[ref-55] Pawlowski J, Bruce K, Panksep K, Aguirre FI, Amalfitano S, Apothéloz-Perret-Gentil L, Baussant T, Bouchez A, Carugati L, Cermakova K, Cordier T, Corinaldesi C, Costa FO, Danovaro R, Dell’Anno A, Duarte S, Eisendle U, Ferrari BJD, Frontalini F, Frühe L, Haegerbaeumer A, Kisand V, Krolicka A, Lanzén A, Leese F, Lejzerowicz F, Lyautey E, Maček I, Sagova-Marečková M, Pearman J, Pochon X, Stoeck T, Vivien R, Weigand A, Fazi S (2022). Environmental DNA metabarcoding for benthic monitoring: a review of sediment sampling and DNA extraction methods. Science of The Total Environment.

[ref-56] Ransome E, Geller JB, Timmers M, Leray M, Mahardini A, Sembiring A, Collins AG, Meyer CP (2017). The importance of standardization for biodiversity comparisons: a case study using autonomous reef monitoring structures (ARMS) and metabarcoding to measure cryptic diversity on Mo’orea coral reefs, French Polynesia. PLOS ONE.

[ref-57] Ratnasingham S, Hebert PDN (2007). BOLD: the barcode of life data system (http://www.barcodinglife.org). Molecular Ecology Notes.

[ref-58] R Core Team (2020). https://www.r-project.org.

[ref-59] Ren W, Zhou P, Zhang D (2025). Assessing the efficiency of COI primers in eDNA metabarcoding for marine metazoan biodiversity. Marine Environmental Research.

[ref-60] Sinniger F, Pawlowski J, Harii S, Gooday AJ, Yamamoto H, Chevaldonné P, Cedhagen T, Carvalho G, Creer S (2016). Worldwide analysis of sedimentary DNA reveals major gaps in taxonomic knowledge of deep-sea benthos. Frontiers in Marine Science.

[ref-61] Steyaert M, Priestley V, Osborne O, Herraiz A, Arnold R, Savolainen V (2020). Advances in metabarcoding techniques bring us closer to reliable monitoring of the marine benthos. Journal of Applied Ecology.

[ref-62] Stoeck T, Bass D, Nebel M, Christen R, Jones MDM, Breiner HW, Richards TA (2010). Multiple marker parallel tag environmental DNA sequencing reveals a highly complex eukaryotic community in marine anoxic water. Molecular Ecology.

[ref-63] Struck T, Purschke G, Halanych K (2006). Phylogeny of Eunicida (Annelida) and exploring data congruence using a Partition Addition Bootstrap Alteration (PABA) approach. Systematic Biology.

[ref-64] Tang CQ, Leasi F, Obertegger U, Kieneke A, Barraclough TG, Fontaneto D (2012). The widely used small subunit 18S rDNA molecule greatly underestimates true diversity in biodiversity surveys of the meiofauna. Proceedings of the National Academy of Sciences of the United States of America.

[ref-65] Tedersoo L, Hosseyni Moghaddam MS, Mikryukov V, Hakimzadeh A, Bahram M, Nilsson RH, Yatsiuk I, Geisen S, Schwelm A, Piwosz K, Prous M, Sildever S, Chmolowska D, Rueckert S, Skaloud P, Laas P, Tines M, Jung JH, Choi JH, Anslan S (2024). EUKARYOME: the rRNA gene reference database for identification of all eukaryotes. Database.

[ref-66] Turon X, Antich A, Palacín C, Præbel K, Wangensteen OS (2020). From metabarcoding to metaphylogeography: separating the wheat from the chaff. Ecological Applications.

[ref-67] Van der Loos LM, Nijland R (2021). Biases in bulk: DNA metabarcoding of marine communities and the methodology involved. Molecular Ecology.

[ref-68] Wangensteen OS, Palacín C, Guardiola M, Turon X (2018). DNA metabarcoding of littoral hardbottom communities: high diversity and database gaps revealed by two molecular markers. PeerJ.

[ref-69] Wickham H (2016).

[ref-70] Willassen E, Westgaard JI, Kongsrud JA, Hanebrekke T, Buhl-Mortensen P, Holte B (2022). Benthic invertebrates in Svalbard fjords—when metabarcoding does not outperform traditional biodiversity assessment. PeerJ.

[ref-71] WoRMS Editorial Board (2025). World register of marine species. https://www.marinespecies.org.

